# Incidence of postoperative nausea and vomiting is not increased by combination of low concentration sevoflurane and propofol compared with propofol alone in patients undergoing laparoscopic gynecological surgery

**DOI:** 10.1186/s40981-019-0292-4

**Published:** 2019-11-02

**Authors:** Yuka Uchinami, Satoshi Takikawa, Fumiki Takashima, Yosuke Maeda, Satoki Nasu, Ayumi Ito, Tatushi Saito

**Affiliations:** Department of Anesthesiology, Hakodate Central General Hospital, 3-2, Honcho3, Hakodate, 040-8585 Japan

**Keywords:** Postoperative nausea and vomiting, Propofol, Sevoflurane, Sevoflurane propofol combination

## Abstract

**Background:**

The incidence of postoperative nausea and vomiting (PONV) is higher in patients receiving volatile anesthetics than those receiving total intravenous anesthesia (TIVA) with propofol. However, it is unclear whether its incidence is increased when a low concentration of sevoflurane is used in combination with propofol.

**Methods:**

This prospective, randomized, controlled trial enrolled women undergoing laparoscopic gynecological surgery. Patients were randomly assigned to receive general anesthesia either with propofol alone (group P) or with 0.8% sevoflurane and propofol (group SP, *n* = 36, each group) for maintenance of anesthesia. The incidence of PONV and the number of patients who required antiemetics were compared.

**Results:**

There were no differences in the incidence of PONV and the number of patients who required antiemetics between the P and SP groups.

**Conclusions:**

A combination of 0.8% sevoflurane and propofol to maintain anesthesia does not increase the incidence of PONV compared with TIVA with propofol.

**Trial registration:**

UMIN-CTR UMIN000023647, registered 14 August 2016.

## Background

Postoperative nausea and vomiting (PONV) is one of the most common adverse effects of anesthesia and is experienced by 25–30% of patients [1]. This affects the recovery process and patient satisfaction following surgery [2, 3]. Several factors, including the patient characteristics (sex, smoking status, history of motion sickness or PONV), anesthetic technique employed, and the type of surgery performed, are known to increase the risk of PONV. Total intravenous anesthesia (TIVA) is one method that can be used to reduce the incidence of PONV compared to inhalational anesthesia [4–8].

The coadministration of propofol with sevoflurane to maintain anesthesia has been suggested recently [9, 10] due to the antiemetic effect of propofol [11], the myocardial protective effects of sevoflurane [12, 13], and the possibility of smooth emergence from anesthesia. It has also been demonstrated that the coadministration of sevoflurane and propofol to maintain anesthesia reduces the incidence of PONV compared with sevoflurane alone [14, 15]. Therefore, propofol with sevoflurane to maintain anesthesia could provide an easy-to-use alternative to using TIVA for people at risk of developing PONV. However, the incidence of PONV using propofol in combination with inhalational anesthesia compared to using TIVA is not known. Therefore, the aim of this study was to compare the incidence of PONV between general anesthesia with propofol alone and propofol in combination with sevoflurane.

## Methods

The study was a prospective, randomized, observer-blinded, interventional, single-center trial, conducted in accordance with the current Declaration of Helsinki. Ethics approval was obtained from the institutional review board of the Hakodate Central Hospital, Hakodate, Japan (April 22, 2016, No. 2016-2). This trial was registered with the UMIN Clinical Trial Registry (UMIN 000023647).

Between August 2016 and February 2017, we enrolled 88 consecutive patients aged 20–60 years, who were scheduled to undergo elective laparoscopic gynecological surgery under combined general/epidural anesthesia. Written informed consent was obtained from all the patients before randomization. The exclusion criteria were patients with ASA physical status ≥ III, with pregnancy or cognitive dysfunction. Patients for whom surgical procedure was changed to laparotomy, epidural anesthesia was ineffective, or follow-up data were lost were also excluded.

After providing patients with an explanation of this study, to evaluate the PONV risk score, patients were asked three questions to ascertain whether they had a history of smoking, motion sickness, or PONV [14]. An affirmative answer for each question was scored 1 point. Subsequently, patients were randomly assigned to two groups using computer-generated random numbers (JMP, version 12.0.1; SAS Institute, Cary, NC, USA). The patient allocation was revealed to each anesthesiologist via a sealed envelope on the day of the surgery. The surgeons, anesthesiologists, statistical analysts, and medical staff in the operating room were not blinded to the treatment group, while the patients, ward staff, and observers were blinded.

No preoperative sedatives or analgesics were administered. After placing the peripheral vein and epidural catheters at the Th12/L1 level, an electrocardiogram, pulse oximetry, noninvasive arterial pressure, muscle relaxant, and end-tidal CO_2_ (EtCO_2_) monitors were used. Propofol was administrated via a target-controlled infusion (TCI) device (Cardinal Health, Basingstoke, UK), using a three-compartment model that was proposed by Schnider et al. [15]. Anesthesia induction was performed using propofol TCI (3–5 μg/ml), remifentanil (0.1–0.5 μg/kg/min), fentanyl (0.2–2 μg/kg), and rocuronium (0.6–0.9 mg/kg). Following tracheal intubation, the lungs were ventilated so as to maintain EtCO_2_ between 35 and 45 mmHg in 50% oxygen at a total flow of 1 L/min. Patients received either propofol alone (group P) or 0.8% sevoflurane and propofol (group SP) for maintenance of anesthesia. In addition to propofol with an infusion rate adjusted to maintain bispectral index (BIS; A-2000TM SP, Aspect Medical System, Norwood, MA, USA) between 40 and 60, i.v. remifentanil 0.05–0.25 g/kg/min and epidural ropivacaine (0.75%, 4–5 ml/h) were administered to all patients. Fentanyl, antiemetics, or nonsteroidal anti-inflammatory drugs were not administered during surgery. Rocuronium 10 mg was administered when train-of-four (TOF) count was > 1. Hypotension (MAP < 60 mmHg) was treated with intravenous phenylephrine or ephedrine.

The administration of propofol, remifentanil, and sevoflurane was stopped upon completion of the surgery. Sugammadex 2 mg/kg (when the TOF count was ≥ 2) or 4 mg/kg (when the TOF count was ≤ 1) was administered. Subsequently, patients were observed without being awoken until they naturally aroused. Tracheal extubation was performed when spontaneous breathing recovered sufficiently with a TOF count of > 0.9, and the patients were able to follow verbal commands.

After operation, 4 ml/h of 0.25% levobupivacaine was administrated via an epidural catheter for postoperative analgesia. Pain assessment was conducted by ward nurses every 3 h, and flurbiprofen or acetaminophen was administered for additional analgesia.

The primary outcome of this study was the incidence of PONV. We also scored the severity of the PONV (0 = no nausea, 1 = mild nausea recovered without antiemetics, 2 = severe nausea requiring antiemetics, and 3 = retching, vomiting, or both) every 3 h until up to 12 h post-surgery and then evaluated the maximum PONV score. We defined patients with a PONV risk score of 1 or less as low risk, and a PONV risk score of 2 or more as high risk, which was used to evaluate the max PONV score. Other factors that were evaluated included the following: level of agitation evaluated using the Ricker sedation scale (1–7 points); amount of coughing at two time points, immediately after extubation and on leaving the operation room [16]; pain scale (0–10) [17]; scores assigned to the quality of recovery assessed via a questionnaire [18]; and perioperative patient satisfaction scores (1 = very unsatisfied, 2 = somewhat unsatisfied, 3 = not satisfied or unsatisfied, 4 = somewhat satisfied, and 5 = very satisfied). These were recorded 24–30 h following the day of surgery.

The sample size was calculated by power analysis while designing the study. We included independent cases and controls, with 1 control per case. A previous study [19] indicated that the incidence of PONV after TIVA was 0.125 and that when using an inhalation agent was 0.415. On applying these results to the sample size calculation, a total of 36 experimental and 36 control subjects were required to reject the null hypothesis, and such that the failure rates for experimental and control subjects are equal with a power of 0.8. The probability of type I error associated with the testing of this null hypothesis was 0.05 with an uncorrected chi-squared test (PS: Power and Sample Size Calculation version 3.1.2, 2014, Vanderbilt University, Nashville, TN, USA).

The values were either expressed as median (interquartile range [IQR]) or by percentage of patients (%). Statistical analyses were performed using commercially available software (JMP, version 12.0.1; SAS Institute, Cary, NC, USA). An independent *t* test was used to analyze parametric data, and nonparametric data were analyzed by the Mann–Whitney *U* test. Categorical variables were evaluated by the Fisher exact test. *P* < 0.05 was considered statistically significant.

## Results

**Fig. 1:**
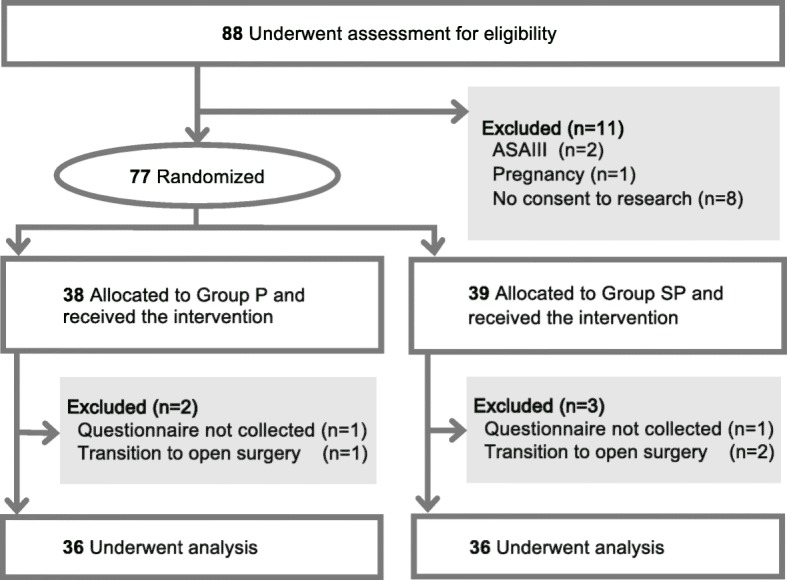
Flow diagram of patients enrolled in the study

**Fig. 2:**
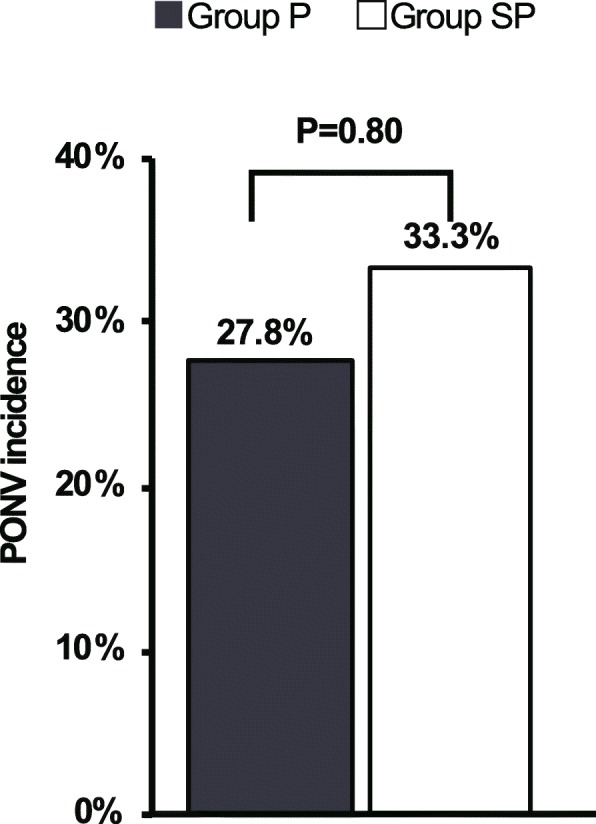
Incidence of postoperative nausea and vomiting. Anesthesia was maintained with propofol (group P) and with a combination of sevoflurane and propofol (group SP)

Among 88 patients eligible for this study, 11 were excluded (2, ASA-PS III; 1, pregnant; 8, did not provide consent). Consequently, 77 patients were randomized and received the intervention. Following this, two patients were excluded because they did not complete the interview form, and three patients were excluded because they were transitioned to open surgery. Eventually, the remaining 36 patients in each group successfully completed the study protocol (Fig. 1). Table 1 shows the demographic data of patients. Patients’ profiles, PONV risk score, and the type of surgery were similar between the two groups. There were no differences in the incidence of PONV (27.8% and 33.3% in the groups P and SP, respectively) (Fig. 2) and the total PONV score (0 [0–1] vs 0 [0–2]; median [interquartile range]) between the two groups. The number of patients who required antiemetics was also not different between the two groups (Table 2). The total doses of rocuronium, fentanyl, and infusion fluid volume were significantly higher in group P than in group SP. No significant differences were recorded between the groups in the pain experienced at the wound site (mainly umbilical site) (median 6, IQR 3–8 vs. median 5, IQR 4–7.75; *P* = 0.685); however, the shoulder pain was significantly more intense in group P than in group SP (median 2.5, IQR 0–5.75 vs. median 0, IQR 0–4; *P* = 0.047) (Table 2).

**Table 1:** Demographic data of patients enrolled in the study

Characteristic	Group P [*N* = 36]	Group SP [*N* = 36]	*P* value
Age (years)	41.8 ± 8.0	40.0 ± 8.7	0.24
Height (cm)	158.7 ± 5.5	157.8 ± 6.2	0.80
Weight (kg)	56.3 ± 7.7	57.4 ± 10.2	0.92
BMI (kg/m^2^)	22.4 ± 3.3	23.0 ± 3.6	0.51
ASA class [*n* (%)]			
I	20 (55.6%)	19 (52.8%)	1.00
II	16 (44.4%)	17 (47.2%)	
PONV risk score	1.1 ± 0.6	1.1 ± 0.7	0.70
Type of surgery (*n*)			
Ovarian cystectomy/tumorectomy	7	17	0.089
Oophorectomy	1	1	
Myomectomy	8	5	
Endometriosis lesion removal	2	2	
Tubalectomy	1	0	
Hysterectomy	16	8	
Adhesion exfoliation	1	3	

Data are presented as mean ± SD or the number of patients and frequency (%). Anesthesia was maintained with propofol (group P) and with a combination of sevoflurane and propofol (group SP)

**Table 2:** Anesthesia-related parameters and recovery profiles

Characteristic	Group P [*N* = 36]	Group SP [*N* = 36]	*P* value
Duration of surgery (min)	155 (110.5, 194.5)	120 (86.5, 219.7)	0.13
Total dose of rocuronium (mg)	100 (70, 163)	80 (60, 97.5)	0.029
Dose of fentanyl (μg)	25 (25, 50)	25 (25.25)	0.0078
Dose of remifentanil per hour (mg/h)	0.0083 (0.0077, 0.013)	0.0092 (0.0071, 0.012)	0.78
Blood loss (ml)	67.5 (10, 145)	50 (10, 75)	0.10
Fluid volume (ml)	700 (512.5, 1012.5)	450 (312.5, 550)	< 0.001
Usage of antiemetics (%)	8 (22.2)	8 (22.2)	1.00
Time to extubation (min)	10 (6, 12)	9 (7, 11)	0.37
Aroused excitement score	4 (3, 4)	4 (3, 4)	0.56
Cough score	0 (0, 0)	0 (0, 1)	0.31
The quality of recovery score	14 (11, 15)	13 (11, 15)	0.39
Satisfaction score	3 (2, 4)	3 (2, 4)	0.72
NRS score of wound sight	6 (3, 8)	5 (4, 7.75)	0.68
NRS score of shoulderpain	2.5 (0, 5.75)	0 (0, 4)	0.030

Data are presented as median (IQR) or the number of patients and frequency (%). Anesthesia was maintained with propofol (Group P) and with a combination of sevoflurane and propofol (Group SP)

## Discussion

We have shown that sevoflurane/propofol combination did not increase the incidence of PONV compared with propofol alone. Liang et al. [20] showed no differences in the use of postoperative antiemetics between patients receiving the combination of sevoflurane/propofol and sevoflurane alone. On the other hand, Kawano et al. [21] showed a lower incidence of PONV in those receiving the sevoflurane/propofol combination than those receiving sevoflurane alone, suggesting the antiemetic effect of propofol. However, there have been no studies comparing the sevoflurane/propofol combination and propofol alone on the development of PONV.

Present findings would result from the antiemetic effect of propofol and low concentration of sevoflurane (0.8%) used with propofol for maintaining the depth of anesthesia compared with sevoflurane alone. Gan et al. [22] found that the plasma propofol concentration associated with a 50% decrease in nausea scores was 343 ng/ml. In the present study, average plasma concentration of propofol was 1.2 μg/ml in patients receiving the combination of propofol/sevoflurane and it requires approximately 30 min to decrease its plasma concentration below 343 ng/ml, suggesting that propofol may have effectively prevented the early stage of PONV. The differences in the dose of fentanyl and fluid volume would not have influenced the incidence of PONV between those two groups of patients.

There were no significant differences in the level of wound pain; in contrast, patients receiving propofol only reported a higher numerical rating scale for shoulder pain. Although the reason is not clear, lower neuromuscular blocking effect of propofol than sevoflurane might account for this because deeper neuromuscular blockades reduce shoulder-tip pain following laparoscopic surgery [23]. Future studies are required to confirm this hypothesis.

There are several limitations to our study. First, we did not administer PONV prophylaxis. This was because we tried to investigate the baseline risk, which could have been masked by prophylactic antiemetics. Especially in the case of high-risk patients, further studies are necessary to compare the results when antiemetics are used. Second, the study subjects were limited only to women. Our aim was to conduct research on relatively high PONV risk patients who were undergoing laparoscopic surgeries and were young women; therefore, it is unclear whether this result can be applied to other patient groups. Third, only one combination of propofol infusion rate and sevoflurane concentration was studied, and the effects of other combinations were not assessed. Therefore, the optimal combination of the propofol infusion rate and sevoflurane concentration remains to be determined.

## Conclusion

In conclusion, general anesthesia maintenance with the coadministration of sevoflurane and propofol does not increase PONV compared to TIVA and, therefore, can be one of the useful methods to maintain general anesthesia. Further studies are needed to show the effectiveness of propofol/sevoflurane combination anesthesia.

## Data Availability

This is available as an electronic file upon request.

## Abbreviations

EtCO_2_: End-tidal CO_2_
NRS: Numerical
PONV: Postoperative nausea and vomiting
TCI: Target-controlled infusion
TIVA: Total intravenous anesthesia
TOF: Train-of-four
